# Primary Vaginal Amelanotic Melanoma: A Diagnostic Conundrum

**DOI:** 10.7759/cureus.20796

**Published:** 2021-12-29

**Authors:** Purwa Patil, Wasif Ali Khan, Vaishali Walke, Ketaki Patil

**Affiliations:** 1 Pathology, Grant Government Medical College and Sir J.J. Group of Hospitals, MUMBAI, IND; 2 Basic Sciences, Almaarefa University, Riyadh, Riyadh, SAU; 3 Pathology, All India Institute of Medical Sciences, Bhopal, IND; 4 Pathology, Bharati Vidyapeeth, Pune, IND

**Keywords:** histological features, vaginal, primary, immunohistochemistry, amelanotic melanoma

## Abstract

Primary vaginal amelanotic melanoma (PVAM) is an exceptionally rare aggressive malignancy having a poor prognosis. PVAM shows a high incidence of recurrence, regional spread, and early metastasis that contribute to a high mortality rate. The majority of primary vaginal malignant melanomas are pigmented, and <10% are amelanotic. Because of the absence of melanin pigment in tumor cells, PVAM may mimic other common vaginal malignancies having a more favorable prognosis and may lead to diagnostic conundrum. Knowledge regarding varied histological features and immunohistochemistry can help in establishing the correct diagnosis. Magnetic resonance imaging (MRI) provides valuable information related to the extent of vaginal malignancy and the involvement of regional lymph nodes. Conservative surgery is used for early-stage disease, whereas advanced stages are treated with radical surgery, chemotherapy, radiotherapy, immunotherapy, and targeted therapy.

## Introduction

Malignant transformation of melanocytes that contain a dark pigment can lead to melanoma. Melanocytes are most commonly present in the epidermis of the skin. In addition, melanocytes are observed in various mucosal sites such as respiratory, gastrointestinal, and urogenital tracts, particularly in the basal portion of the vaginal epidermis in 3% of healthy women [1,2]. Hence, the skin is the most common site of melanoma, with cutaneous melanoma (CM) being the 19th most common cancer worldwide [3]. The vagina is a rare site for the origin of mucosal melanoma (MM) and accounts for only 0.3%-0.8% and, in some studies, up to 1.4% of all melanomas [4,5]. MMs are considerably more common in women than in men. Primary vaginal malignant melanoma (PVMM) usually develops in the lower third and is mostly present as either inconsistently pigmented grey or black nodular, fungating or polypoid soft mass ranging from 0.5 to 0.8 cm in size in the anterior wall of the vagina [5,6]. Most patients with PVMM present with irregular vaginal bleeding or increased discharge from the vagina. Some patients may present with a gradually enlarging vaginal wall mass or dyspareunia [5,6]. PVMM commonly occurs in postmenopausal women, with an average age at diagnosis of 68 years (wide age range of 28-100 years). In contrast to CM, PVVM is considerably aggressive in nature and has poor prognosis, and the five-year overall survival (OS) rate is 5%-32.3% [7]. Malignant melanoma can be subdivided into five types: superficially spreading melanoma, nodular melanoma, lentigo maligna, acral lentiginous melanoma, and amelanotic melanoma [8]. The majority of PVMMs are pigmented, and <10% are amelanotic [4]. Lack of the melanin pigment in primary vaginal amelanotic melanoma (PVAM) and the presence of a mixed histological pattern create diagnostic conundrum and may lead to misdiagnosis.

## Case presentation

**Figure 1:**
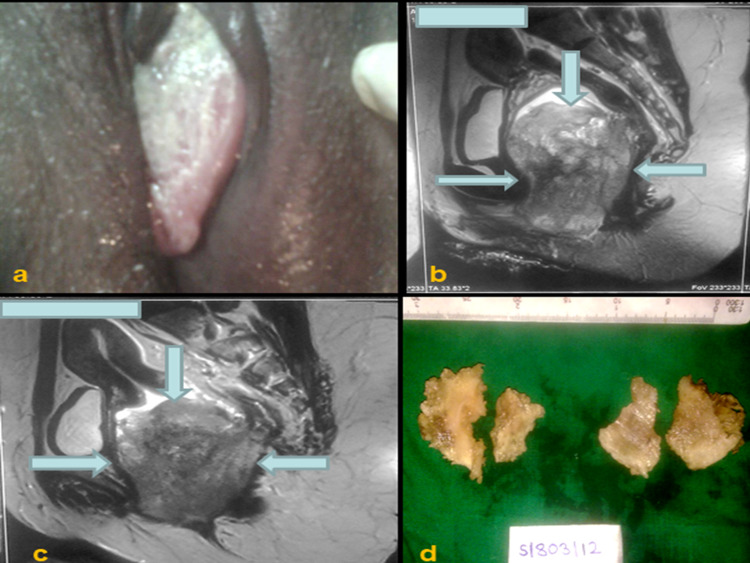
(a) Clinical image showing non-pigmented polypoid tumor arising from the lower third of the vagina and involving posterior and lateral wall. (b and c). MRI shows a large heterogenous hyperintense lesion at the vaginal and cervical region in T2-weighted images. (d) Gross showing non-pigmented grayish-white tissue biopsy pieces.

**Figure 2:**
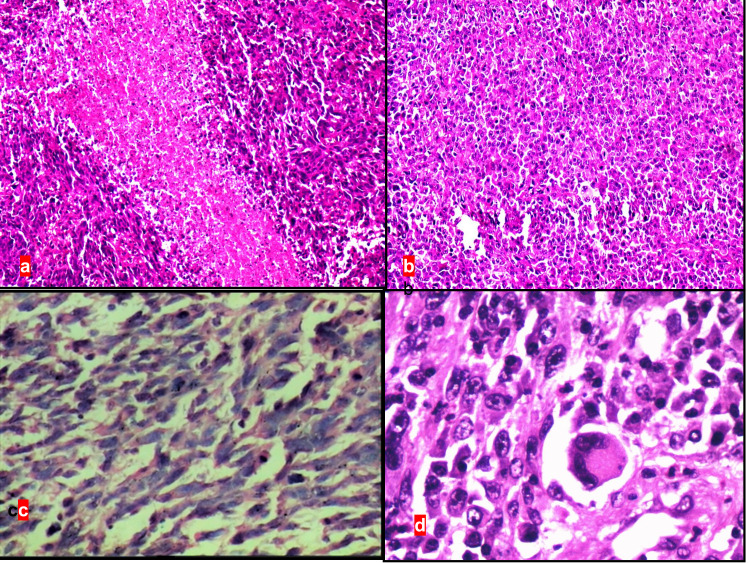
(a) H&E (x40) stained section showing mixed pattern comprising of epithelioid to spindle-shaped tumor cells arranged in sheets with large areas of necrosis. (b) H&E (x40) stained section showing epithelioid tumor cells arranged in sheets. (c) H&E (x100) stained section showing spindle-shaped tumor cells arranged in sheets. (d) H&E (x400) stained section showing marked nuclear pleomorphism, hyperchromatism, irregular nuclear membranes, and prominent nucleoli. Melanin pigment is notably absent. Multinucleate giant cell is also noted. H&E, hematoxylin and eosin

**Figure 3:**
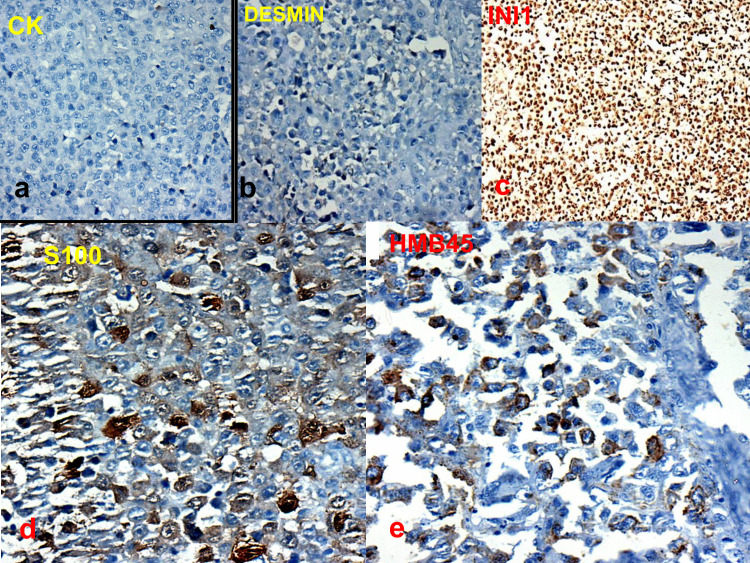
(a) Photomicrograph (immunostain, x100) reveals negative cytokeratin (CK). (b) Photomicrograph (immunostain, desmin, x100) showing absence of immunoreactivity. (c) Photomicrograph (immunostain, INI1, x100) showing immunopositive tumor cells. (d) Photomicrograph (immunostain, x400) reveals S100 positivity. (e) Photomicrograph (immunostain, HMB45, x400) reveals immunopositivity.

A 43-year-old woman presented with a three-month history of amenorrhea, foul-smelling vaginal discharge, and pain in the right hip joint. Gynecological examination revealed a large polypoid growth of size 4.5 x 3 x 3 cm arising from the lower third of the vagina and involving the posterior and lateral wall (Figure 1a). Thickening of the bilateral parametria was observed. Rectal examination showed the presence of a firm mass on the anterior wall of the rectum. Magnetic resonance imaging (MRI) of the pelvis revealed a large heterogenous hyperintense lesion at the vaginal and cervical region on T2-weighted images. Anterior fat planes were maintained with no evidence of invasion of the urinary bladder, whereas posterior fat planes and anterior rectal wall appeared to be involved by the tumor (Figures 1b, 1c). Subsequently, incisional biopsy was performed. Gross examination showed a grayish white, non-pigmented tissue piece of size ranging from 1.5 x 0.5 to 0.5 x 0.5 cm (Figure 1d). Histopathological examination revealed a mixed pattern comprising epithelioid to spindle-shaped cells arranged in sheets with large areas of necrosis. The nuclei of tumor cells showed pleomorphism, hyperchromatism, irregular nuclear membranes, and prominent nucleoli. The melanin pigment was notably absent. In addition, binucleate and multinucleate giant cells were observed (Figures 2a-2d). Following tentative differential diagnosis was signed out: (1) poorly differentiated squamous cell carcinoma/sarcomatoid carcinoma and (2) sarcoma. Subsequently, immunohistochemistry (IHC) was performed using cytokeratin (CK), desmin, and INI-1 markers. The findings for CK and desmin were negative. However, INI-1 was extensively expressed by tumor cells. Thus, we examined S-100 and human melanoma black-45 (HMB45) markers, which showed strong positivity (Figures 3a-3e). Clinical examination of the skin, mucosa, and eyes did not reveal any suspicious pigmented lesions, thus ruling out the possibility of metastatic melanoma to the vaginal wall. An American Joint Committee on Cancer (AJCC), 8th Edition, staging system for vaginal MM is not available [6]. A final diagnosis of PVAM was established.

## Discussion

The tumourigenesis of vaginal melanoma involves NRAS mutations ranging from 13% to 43% and C-KIT amplifications ranging from 0% to 13% [6,9,10]. PVAM should be diagnosed as early as possible because it is an aggressive malignancy that spreads and metastasizes in the early course of the disease and has a considerably high mortality rate. MRI examination provides valuable information related to the extent of the vaginal malignancy and the involvement of regional lymph nodes [7]. However, histological examination through biopsy, followed by IHC, is the mainstay in the diagnosis of vaginal malignancies. Histologically, melanomas can be classified into epithelioid (55%), mixed (28%), and spindle (17%) subtypes [11]. Lack of the melanin pigment, as observed in the case of amelanotic melanoma, and varied histological features create diagnostic difficulty, thus resulting in a wide-spectrum differential diagnoses including poorly differentiated squamous cell carcinoma, sarcomatoid carcinoma, adenocarcinoma, leiomyosarcoma, and epithelioid sarcoma [12,13]. Ancillary techniques such as IHC play a crucial role in establishing the correct diagnosis. Immunohistochemically, melanomas are positive for S-100, melan A, and HMB45. S-100 is expressed by almost all primary and metastatic melanomas. HMG45 has the highest specificity and melan A has the highest sensitivity in diagnosing melanomas. Negative CK and desmin exclude carcinomas or sarcomatoid carcinomas and leiomyosarcoma, respectively. Loss of INI1/SMARCB1 expression is observed in epithelioid sarcomas, in contrast to the majority of melanomas that show immunopositivity [6,14,15].

MMs spread locally and metastasize early, which can be explained by the extensive vascular and lymphatic drainage of the vaginal mucosa. MMs recur locally, metastasize to the lymph nodes and viscera, and can even cause life-threatening hemorrhage. The vagina, vulva, and groin are the common sites of recurrence, whereas the lungs, liver, bones, and brain are the common distant metastatic sites. The most vital prognostic factor is a tumor size of <3 cm [13]. MRI evaluation is necessary for staging, deciding the operative strategy, and following up patients. The median OS duration was 41 months in patients with a tumor size of <3 cm but only 12 months in patients with larger tumors [6]. Despite treatment, the five-year survival rate was reported to be less than 30% [16].

Surgery is the preferred treatment modality in the case of early-stage resectable tumors. Other modalities include chemotherapy, radiotherapy, immunotherapy, and targeted therapy.

Wide local excision (WLE) with a safety margin of 1 cm (Breslow tumor depth of 2 mm or less) or WLE with a safety margin of 2 cm along with radiotherapy (Breslow tumor depth of 2 mm or more) is the treatment of choice in the early stage. An aggressive treatment strategy in the form of radical surgery (vaginectomy to pelvic exenteration) and lymphadenectomy with adjuvant chemotherapy or radiotherapy is reserved for advanced stages. Chemotherapy is administered in the form of dacarbazine, nitrosourea, and imatinib either alone or in combination. Recently, for unresectable or metastasized tumors, immunotherapy in the form of interferon alpha-2b has been approved by the U.S. Food and Drug Administration. Targeted therapy using monoclonal antibodies such as ipilimumab and nivolumab is a novel treatment indicated for some unresectable tumors [14].

## Conclusions

To summarize, PVAM is an extremely uncommon but aggressive malignancy having poor prognosis. Histological features and the absence of the melanin pigment may create a diagnostic difficulty. Although rare, PVAM should be considered as one of the differential diagnoses in the evaluation of vaginal malignancies. IHC plays a crucial role in confirming the diagnosis. Early-stage disease is treated with conservative surgery, whereas radical surgery is recommended for advanced malignancies.
